# Knowledge and attitudes of medical students toward death: a cross-sectional comparative study between an Arab and a Western University

**DOI:** 10.1186/s40359-024-01616-w

**Published:** 2024-03-08

**Authors:** Randah R. Hamadeh, Izzeldin Abuelaish, Susan J. Yousufzai, Yousef AT AlShammari, Yomna E. Ahmed, Haitham A. Jahrami

**Affiliations:** 1 College of Medicine and Medical Sciences, Arabian Gulf University, Manama, P.O. Box: 26671, Bahrain; 2 Dalla Lana School of Public Health, University of Toronto, Toronto, Canada; 3 Temerty Faculty of Medicine, University of Toronto, Toronto, Canada; 4 Government Hospitals, Manama, Bahrain

**Keywords:** Bereavement, Cultures, Death, Dying, Grief, Medical students, Religiosity

## Abstract

**Background:**

Cultural factors influence attitudes toward death, and gender disparities are evident. Prior studies show that medical students have limited knowledge about death and are uncomfortable with it. Moreover, there is limited research that has examined factors that influence medical students’ knowledge and attitudes toward death.

**Objectives:**

The objectives of the study were to compare cultural and gender differences in relation to knowledge and attitudes toward loss and grief and to screen for complicated grief among medical students at the Arabian Gulf University and the University of Toronto.

**Methods:**

A cross-sectional study was disseminated to medical students at both universities in 2022. The variables in the survey included four parts: demographic characteristics of the participants, religious observance, history of encountering loss of a loved one, grief following loss, attitude toward death, and learning about how to deal with grief and death during medical school. The brief grief questionnaire and the death attitude profile-revised scales were used.

**Results:**

The study sample consisted of 168 medical students, with 74.1% being female. Complicated grief scores were higher among Arabian Gulf University students (3.87 ± 2.39) than among University of Toronto students (2.00 ± 1.93) and were higher for participants with a higher degree of religious observance in both schools *(p* < 0.05). Death avoidance (*p* = 0.003), approach acceptance (*p* < 0.001), and escape acceptance (*p* = 0.038) domains were significantly higher among Arabian Gulf University students than among University of Toronto students. Almost three-quarters of University of Toronto students reported not being taught about grief, compared to 54% of Arabian Gulf University students.

**Conclusions:**

Arabian Gulf University medical students scored higher on complicated grief, most likely due to cultural and religious factors. Females at both institutions as well as those who indicated a higher level of religious observance reported higher scores of complicated grief. The study highlights how cultural and religious beliefs influence medical students’ attitudes toward death and bereavement. It provides valuable insight into the knowledge and attitudes of medical students toward loss.

**Supplementary Information:**

The online version contains supplementary material available at 10.1186/s40359-024-01616-w.

## Background

Medical students interact with patients on a regular basis, which can include those facing serious illnesses and death [1]. It is therefore important to understand how medical students perceive, understand, and respond to death, both in the present and in their future work as medical practitioners [2].

Studies have revealed that medical students have a limited knowledge base of death-related topics and attitudes toward death [3–6]. Furthermore, medical students tend to try to distance themselves from death, leading to a lack of empathy and understanding among them [4]. To address this issue, medical schools have begun to implement death education initiatives [5]. These initiatives provide students with the opportunity to gain a better understanding of death-related topics and learn how to appropriately respond in different healthcare settings when dealing with death [6]. The educational material focuses on topics such as hospice care, palliative care, bereavement, euthanasia, and others, allowing students to gain knowledge about the subject [7].

In addition to formal death education initiatives, medical schools are also taking proactive steps to promote an open dialog regarding death [8, 9]. This includes providing students with the time and space to express their feelings and ask questions in a safe, secure, and confidential environment [10]. These open conversations allow students to better understand death, which can improve their knowledge and attitudes toward those facing death [11].

As we explore the attitudes of medical students toward death, research studies have revealed some interesting findings toward this topic [12, 13]. This information can be used to inform curriculum design and teaching practices to ensure that students become adequately educated and well prepared to address matters related to death [14]. Medical students have a strong awareness of death, with the majority reporting having had prior exposure to death through the loss of a family member or friend [13]. These experiences have led students to become more knowledgeable about death, with many showing an interest in understanding its social, religious, psychological, and cultural aspects [15]. This implies that, as medical professionals, these students may be more likely to approach death with empathy, compassion, and respect [16].

At the same time, medical students still have some misconceptions and fears toward death, which can be due to a lack of knowledge, cultural beliefs, and negative experiences [17]. For example, some medical students might lack an understanding of death’s inevitability as a natural process or view death as a failure of medical science rather than a natural event [18]. Therefore, it is important for them to learn about death in a way that informs and corrects these attitudes [19].

Along with attitudes, students’ behavior toward death can affect the quality of care they provide to patients nearing the end of life [20]. Studies indicate that medical students are likely to feel uncomfortable or anxious when facing death and will practice avoidance behaviors such as avoiding the patient rooms or closing the curtains [21]. This can be a challenge when death is a part of the medical profession, and medical students must be trained on how to handle and manage these emotions [22]. Correspondingly, appropriate interventions are lacking for medical students dealing with patient deaths or those who have experienced death in their personal lives [23]. Specifically, evidence suggests that medical curricula are deficient in matters related to death and dying, views on death, communicating sad news, spirituality, and bereavement [24]. Although students may have ‘medical knowledge’ and are expected to be prepared to face loss, there is still a need for emotional support provided through their medical curriculum, as they are often inadequately supported around the time of a patient’s death [25]. Thus, teaching in medical schools about death and bereavement does not seem to be sufficient to prepare future doctors for such inevitable experiences. While research urges the importance of shedding light on the mental health of medical students and the need for access to mental health services with evidence-based treatments [26], there is limited research examining factors that may increase medical students’ susceptibility to psychiatric morbidity and burnout, such as dealing with death. A lack of dialog about the death and dying of patients creates vulnerability among medical students and increases susceptibility to unhealthy means of overcoming failure and sadness [27]. Globally, research shows that medical students are prone to psychiatric morbidity, psychological stress, and burnout [26]. Thus, there is a significant need for support to mitigate such consequences when compounded by adverse experiences regarding the death of a loved one in their personal lives.

Cultural factors play a significant role in influencing attitudes toward mental illness. Medical students experience psychological distress; however, they avoid discussing it due to perceived notions of weakness and incapability of withstanding the arduous academic curriculum, thus they may be prone to suppressing their feelings [26]. Research shows that many medical students struggle with a lack of support and guidance from their seniors and faculty and do not feel comfortable approaching their superiors for help. Diverse reasons include the fear of being burdensome, feelings of awkwardness, the desire to appear professional, the medical team’s insensitivity or lack of emotion, and their disagreement with advice proffered by their seniors to simply desensitize themselves to death [6]. The literature has reported gender disparities in loss among university students. Women were more likely to acknowledge death than men [28] and exhibit a greater degree of apprehension toward death [29]. Female medical students showed great receptiveness to learning about death [28, 30].

The Arabian Gulf University (AGU) is a regional university in Bahrain that offers a medical degree program in 6 years, where students enter their first medical year after completing high school. Most of the students are Gulf Cooperation Council (GCC) citizens sponsored by their countries, and the rest are private GCC citizens or residents. The AGU medical school program, which is taught in English, is designed to provide students with medical knowledge and skills and a thorough understanding of medical ethics and professionalism through problem-based learning (PBL) that progressively builds on the knowledge of previous years and stresses the importance of self-learning. The medical years at AGU are divided into three phases: basic sciences (year 1), medical sciences (years 2–4), and clerkships (years 5–6). On the other hand, the University of Toronto (UofT) is an international university in Canada that accepts students from diverse backgrounds. The medical program at UofT is a PBL 4-year program, taught in English; the first two are foundation years, and the last are clerkships. Admission requirements are the completion of three years of undergraduate study for domestic students or a bachelor’s degree for international students. The two universities are similar in that they participate in PBL. As opposed to UofT, where students’ mother tongue is diverse, that of AGU students is Arabic.

The objectives of the study were to compare cultural and gender differences in relation to knowledge and attitudes toward bereavement and grief, screen for complicated grief (CG), and compare those who experienced theloss of a loved one with those who did not among medical students from AGU and UofT. We hypothesized that medical students from AGU and UofT would differ in their knowledge and attitudes toward death and complicated grief.

## Methods

### Study design

This observational study was structured and reported following the Strengthening the Reporting of Observational Studies in Epidemiology (STROBE) guidelines. The STROBE checklist version of cross-sectional studies was followed. By adhering to the STROBE guidelines, we aim to ensure the research methodology and results are reported with adequate clarity, comprehensiveness, and rigor. The completed STROBE checklist has been included as a supplementary document to highlight our compliance with these important reporting guidelines. See supplemental 1.

### Setting and participants

All year 5–6 medical students at AGU and years 1–4 medical students at UofT who were enrolled during the academic year 2021–2022 and were between 20 and 30 years old made up the study population. Students in years 5 and 6 at AGU are expected to be between 21 and 30 years old, as they begin medical school at the age of 18, whereas students at UofT begin medical school between 21 and 22 years old. This criterion for the age group 20–30 years was put in place so that students from AGU and UofT were comparable in age, which might have comprised most years 5 and 6 medical students from AGU and the student population from years 1–4 from UofT. In addition, given the differences in clerkship years between AGU (years 5 and 6) and UofT (years 3 and 4) curricula, we included in our criteria medical students in years 1–4 from UofT. A total of 81 students were collected from AGU and 90 students from UofT. Three cases were excluded from UofT since they did not report details related to age, year of study, or gender.

The target sample size of 160–180 medical students (80–90 from AGU and 80–90 from UofT) was determined to provide sufficient power to detect meaningful differences in the primary outcome measures between the two groups. Power analyses indicated this sample size would allow detection of a medium effect size (d = 0.5) in the primary outcomes of interest, with 80% power and an alpha of 0.05. This sample provided over 90% power to detect large differences between groups (d ≥ 0.8). The sample was adequately powered for assessing both between-group differences and within-group correlates. While a larger sample could increase statistical power and precision, feasibility constraints limited further enrollment. The achieved sample covered a diverse cross-section of medical students in the specified programs and years of study. We deemed the final sample size adequate to address the study’s objectives while balancing considerations of power, resources, and generalizability.

Both AGU and UofT medical students in the pre-clerkship phase are introduced to death and dying through medical problems, family studies, and professional skills. They are also familiarized with breaking bad news, death and dying, and medical ethics. In the clerkship, during their clinical training, particularly in the psychiatry rotation, they learn more about dealing with dying patients and breaking bad news. They might also witness the death of a patient in their clinical rotations.

A survey was disseminated to medical school students from AGU and UofT in English. Google Forms were sent to the emails of AGU medical school students by one of the AGU investigators after obtaining their emails from the Registrar’s Office. The administrative program at UofT was contacted to disseminate an online survey hosted on Research Electronic Data Capture (REDCap). The survey took approximately 20 min to complete. UofT medical students received an incentive in the form of a $15 gift card. All study procedures were approved by the Research and Ethics Committee at the College of Medicine and Medical Sciences, AGU, and the Ethics Committee at UofT. A consent form was distributed with the questionnaire, informing the students that the survey was anonymous and that their completion of the questionnaire meant that they agreed to participate in the study and that they could withdraw at any part of the survey without being affected.

### Data sources/measurement

The variables in the survey included four parts: demographic characteristics of the participants (sex, age, and ethnicity), religious observance, history of encountering loss of a loved one [e.g., immediate family member (parent, sibling), other relative (e.g., uncle, cousin), grandparent, friend/colleague/other], grief following loss, attitude toward death, and learning about how to deal with grief and death during medical school.

The Brief Grief Questionnaire (BGQ) was used to measure how much the student was affected by the loss of a loved one. It is a 5-item self-report instrument for screening for CG, each of which is scored from 0 to 2 (0 = not at all, 1 = somewhat, 2 = a lot). The BGQ total scores range from 0 to 10. A score of 4 or more suggests that an individual may have CG [31]. The literature documents the validity and reliability of the scale with help-seeking sample populations [32], with a sufficiently high Cronbach’s alpha (= 0.75). The BGQ was included in this study because personal experiences with grief and loss can significantly influence healthcare professionals’ approaches, attitudes, and communication skills when dealing with patient death and bereavement. As future physicians, medical students’ personal grief reactions can shape their clinical preparedness and capacity for providing compassionate end-of-life care. For example, high levels of prolonged or CG may cause distress that impedes students’ ability to engage sensitively with patients. Conversely, resolved grief could enhance empathy. Assessing students’ personal grief responses provides insight into an important factor that may impact their developing perspectives on death and dying. The BGQ helps characterize where students are in the grief process and how this relates to other death-related knowledge, attitudes, and needs assessed in our survey.

Attitude toward death was measured using the Death Attitude Profile —Revised (DAP-R) [33]. This scale consists of 32 items using a 7-item Likert scale, where the attitudes are divided into the following dimensions: Fear of death (7 items) encompasses personal fear of death as well as fear of the death of others; neutral acceptance (5 items) assesses how well a person accepts the reality of death in a natural way, without fearing or welcoming it; approach acceptance (10 items) analyzes the possibility of death as an alternative to a miserable existence; Escape acceptance (5 items) assesses the possibility of death as an alternative to a miserable life, and Death avoidance (5 items) assesses attempts to avoid thinking about death. Scores for all items are from 1 to 7 in the direction of strongly disagree (1) to strongly agree (7). The DAP-R showed adequate validity and reliability, apart from Braun’s low alpha for neutral acceptance [33, 34].

### Analyses

Descriptive statistics, including frequencies and percentages, were conducted to display the characteristics of the study population. Chi-square tests were performed to determine differences between the characteristics of all categorical variables in each sample. Independent samples *t* tests, Mann‒Whitney U tests, and ANOVAs (Analysis of Variance) were conducted to determine the differences in mean (Standard deviation) scores of the BGQ and death attitude scale in relation to each group of medical students (AGU vs. UofT) and all demographic characteristics (sex, relation to the deceased, religious observance, and time passed since death). BGQ characteristics were investigated by calculating item means and standard deviations. The death attitude mean scores were calculated for the total scale and each domain by dividing the total scale score by the number of items forming each scale. Cronbach’s alpha coefficient and item-total correlations were examined to assess the reliability of both scales. Data from the open-ended responses wherein students were asked to specify the courses in which their medical curriculum included teaching about bereavement and grief and to describe what was taught were analyzed using content analysis. Statistical analyses were conducted using the Statistical Package for the Social Sciences, version 28, with a *p*-value < 0.05 considered statistically significant for all tests.

## Results

### Participants

The total study sample consisted of 168 medical school students (AGU = 81 and UofT = 87). The sample population consisted 42% of year-5 medical students at AGU and 58% of year-6 students. The UofT group consisted of students in years 1 (40%), 2 (30%), 3 (14%), and 4 (16%). All AGU students were of Middle Eastern ethnicity, while those of the population from UofT varied [Asian (48%), European (12%), North American (18%), Middle Eastern (1%), African (5%), and mixed (1%)].

Most of the populations in AGU and UofT were females (74% and 81%, respectively). AGU and UofT students were similar in age, with most (AGU 59.3%, UofT 59.8%) being in the age group ≥ 25 years. Half of the total sample showed religious observance, with more AGU students (77%) than UofT students (25%), *χ2* (2) = 46.00, *p* < 0.00001 (Table 1).

**Table 1:** Demographic characteristics of AGU and UofT students

Characteristics	AGU (*n* = 81)	UofT (*n* = 87)	*P* value
	*n* (%)	*n* (%)	
Gender			
Male	21 (25.9)	17 (19.5)	0.3223
Female	60 (74.1)	70 (80.5)	
Age			
< 25	33 (40.7)	35 (40.2)	0 0.946
≥ 25	48 (59.3)	52 (59.8)	
Knowledge about death/grief in medical school			
Yes	37 (45.7)	23 (26.4)	**0.009**
No	44 (54.3)	64 (73.6)	
Degree of religious observance			
Yes	62 (76.5)	22 (25.3)	**< 0.00001**
To some degree	14 (17.3)	34 (39.1)	
No	5 (6.2)	31(35.6)	
Experienced loss of a loved one			
Yes	53 (65.4)	62 (71.3)	0 0.416
No	28 (34.6)	25 (28.7)	

Note. ^a^*x*^*2*^ Test, Bold numbers are statistically significant

### Experience of loss

69% of participants stated that they had experienced the death of a loved one (AGU, 65%; UofT, 71%). Among medical students from AGU (*n* = 53) and UofT (*n* = 62), 87% and 82%, respectively, stated that they had lost a relative (i.e., parent, grandparent, sibling, cousin, uncle) compared to a nonrelative (i.e., friend, colleague, neighbor). The majority (68%) of deaths (AGU, 66%; UofT, (69%) occurred due to noncommunicable diseases (e.g., heart disease, cancer, cerebrovascular disease, kidney disease) (*χ2* (2) = 12.20, *p* = 0.002). The amount of time elapsed between bereavement and survey completion was reported more than a year ago by 77% of participants from AGU and 81% of participants from UofT (Table 2).

**Table 2:** Characteristics of the deceased

Variables	AGU (*n* = 53) *n* (%)	UofT (*n* = 62) *n* (%)	*P* value^a^
Relation to deceased			
Immediate family member	2 (3.8)	13 (21.0)	**0.030**
Grandparent	30 (56.6)	24 (38.7)	
Other relative (unspecified)	14 (26.4)	14 (22.6)	
Friend/colleague/other	7 (13.2)	11 (17.7)	
Cause of death			
Trauma/accidental/nonaccidental injuries	5 (9.5)	16 (25.8)	**0.002**
Noncommunicable/communicable diseases	35 (66.0)	43 (69.4)	
Unspecified/unknown	13 (24.5)	3 (4.8)	
Time passed since death			
≤1 year	12 (23.1)	12 (19.4)	0.627
>1 year	40 (76.9)	50 (80.6)	
Age at death			
≤ 20	6 (11.5)	6 (9.8)	
21–40	7 (13.5)	7 (11.5)	0.962
41–60	8 (15.4)	11 (18.0)	
> 60	31 (59.6)	37 (60.7)	

Note. Missing data: Time since death AGU (*n* = 1); Age of death (*n* = 2). ^a^*x*^*2*^ Test. Bold numbers are statistically significant

### Complicated grief

In this study, the alpha level for the BGQ scale was 0.78. The average CG total scores were significantly higher for AGU medical students than for UofT medical students (AGU = 3.87 ± 2.39, UofT = 2.00 ± 1.93; *t* (113)* = *4.63, *p* < 0.001) (Table 3). The prevalence of CG in the total sample, based on a score of four or more, was 36%. Out of the participants from AGU that reported the loss of a loved one, 57% had a score that suggested they may have CG, compared to 17% of UofT participants (*χ2* (1) = 18.81, *p* < 0.001).

**Table 3:** Complicated grief scores of participants who experienced loss of a loved one by demographic characteristics

Variables	AGU	UofT	*P* value^a^
	M (SD)	M (SD)	
Gender			
Male	3.67 (2.29)	1.00 (1.00)	**0.001**
Female	3.95 (2.45)	2.27 (2.04)	**< 0.0001**
Both genders	3.87 (2.39)	2.00 (1.93)	**< 0.001**
Age			
< 25	3.92 (2.43)	2.29 (2.19)	**0.023**
≥ 25	3.83 (2.39)	1.85 (1.79)	**< 0.001**
Religious observance			
Yes	3.93 (2.31)	1.94 (1.95)	**0.003**
To some degree/	4.67 (2.94)	1.88 (1.70)	**0.005**
No	2.00 (1.83)	2.18 (2.22)	0.879
Relation to deceased			
Relative	4.13 (2.29)	1.96 (1.96)	**< 0.001**
Nonrelative	2.00 (1.82)	2.18 (2.22)	0.867
Time passed since death			
≤1 year	3.92 (2.23)	2.33 (2.19)	0.093
>1 year	3.85 (2.49)	1.92 (1.88)	**< 0.001**
Age at death			
≤ 20	3.00 (1.89)	2.83 (2.14)	0.889
21–40	4.43 (2.44)	1.86 (2.41)	0.071
41–60	4.88 (2.53)	3.64 (2.06)	0.256
> 60	3.65 (2.46)	1.46 (1.48)	**< 0.001**

Note: ^a^Two-sided *p* values reported, Mann‒Whitney U. Bold numbers are statistically significant

CG scores in relation to demographic characteristic comparisons between AGU and UofT showed significant differences. AGU scored higher on CG in relation to all characteristics. AGU males and females scored significantly higher on CG compared to UofT males (*U* = 27.0, z = -3.29, *p* = 0.001) and females (*t* (85) = 3.49, *p* < 0.001). Furthermore, females in both universities had higher CG scores than males. AGU students also scored higher on CG in relation to each age group < 25 (*t*(43) = 2.35, *p* = 0.023) and ≥ 25 (*t*(68) = 3.94, *p* < 0.001), reporting yes to religious observance, (*t*(57) = 3.06, *p* = 0.003), and to some degree, *t*(28) = 3.09, *p* = 0.005). Furthermore, AGU scored higher for losing a relative (*t*(95) = 5.03, *p* < 0.001), time passed since death more than a year ago (*t*(88) = 4.19, *p* < 0.001), and losing someone > 60 years old (*U* = 261.0, z = -3.91, *p* < 0.001).

### Death attitudes

The internal consistency using Cronbach’s alpha for the five subscales in our tool ranged from a low of 0.77 to a high of 0.95: fear of death (0.82), death avoidance (0.89), neutral acceptance (0.77), approach acceptance (0.95), and escape acceptance (0.90). The consistency measures aligned with the original measure, demonstrating good to very good reliability (Wong et al., 1995).

The Death Avoidance domain (t (165) = 3.00, *p* = 0.003), Approach Acceptance (t (165) = 7.65, *p* < 0.001), and Escape Acceptance (t (151.73) = 2.07, *p* = 0.040) domain scores were significantly higher for AGU participants than for UofT participants. Table 4 shows the item means and standard deviations for the five dimensions of the death attitude scale for each group in relation to various characteristics presented in this study.

**Table 4:** Death attitude scale scores of medical students according to various characteristics

Variables	Fear of Death			Death Avoidance			Neutral Acceptance			Approach Acceptance			Escape Acceptance		
	AGU	UofT	*p*-value	AGU	UofT	*p*-value	AGU	UofT	*p*-value	AGU	UofT	*p*-value	AGU	UofT	*p*-value
	M (SD)			M (SD)			M (SD)			M (SD)			M (SD)		
Total	3.96 (1.25)	4.13 (1.27)	0.375	3.78 (1.61)	3.09 (1.33)	**0.003**	5.35 (1.22)	5.37 (0.94)	0.889	4.96 (1.38)	3.34 (1.37)	**< 0.001**	3.48 (1.69)	2.99 (1.32)	**0.040**
Gender															
Male	3.67 (1.12)	3.54 (1.34)	0.736	3.08 (1.58)	2.92 (1.33)	0.744	5.29 (1.25)	5.33 (1.22)	0.914	4.53 (1.43)	3.35 (1.32)	**0.013**	3.27 (1.33)	2.86 (1.34)	0.355
Female	4.06 (1.28)	4.28 (1.24)	0.339	4.02 (1.56)	3.13 (1.35)	**< 0.001**	5.37 (1.22)	5.38 (0.88)	0.947	5.11 (1.34)	3.33 (1.39)	**< 0.001**	3.56 (1.79)	3.03 (1.33)	0.060
Age															
< 25	3.82 (1.13)	4.25 (1.13)	0.126	3.62 (1.58)	3.29 (1.29)	0.353	5.44 (1.16)	5.19 (0.94)	0.343	5.02 (1.55)	3.52 (1.08)	**< 0.001**	3.63 (1.59)	2.65 (0.952)	**0.009** ^†^
≥ 25	4.06 (1.32)	4.06 (1.38)	0.993	3.88 (1.64)	2.95 (1.37)	**0.003**	5.28 (1.26)	5.48 (0.950)	0.367	4.92 (1.26)	3.21 (1.54)	**< 0.001**	3.38 (1.76)	3.24 (1.49)	0.677
Religious observance															
Yes	3.95 (1.26)	4.34 (1.23)	0.205	3.93 (1.59)	3.25 (1.46)	0.085	5.33 (1.10)	5.38 (0.94)	0.833	5.24 (1.19)	4.62 (1.37)	**0.048**	3.29 (1.67)	3.27 (1.44)	0.948
To some degree	4.07 (1.39)	4.04 (1.28)	0.941	3.63 (1.75)	3.08 (1.36)	0.246	5.59 (1.51)	5.26 (0.944)	0.373	4.52 (1.51)	3.22 (1.06)	**0.001**	3.76 (1.73)	2.92 (1.14)	0.056
No	3.83 (0.69)	4.09 (1.36)	0.681	2.32 (0.782)	2.98 (1.28)	0.272	4.96 (1.79)	5.48 (0.982)	0.554	2.78 (1.02)	2.55 (0.97)	0.630	5.04 (0.80)	2.88 (1.45)	**0.003**
Loss of a loved one															
Yes	3.95 (1.29)	4.08 (1.25)	0.591	3.74 (1.65)	2.97 (1.33)	**0.007**	5.32 (1.17)	5.45 (0.97)	0.501	5.04 (1.29)	3.24 (1.42)	**< 0.001**	3.56 (1.65)	2.99 (1.46)	0.057
No	3.98 (1.18)	4.28 (1.39)	0.404	3.84 (1.57)	3.39 (1.35)	0.276	5.41 (1.31)	5.16 (0.89)	0.440	4.81 (1.55)	3.56 (1.24)	**0.002**	3.33 (1.77)	2.98 (0.95)	0.377
Relation to deceased															
Relative	3.91 (1.26)	3.96 (1.23)	0.850	3.76 (1.64)	2.88 (1.37)	**0.005**	5.27 (1.19)	5.49 (0.905)	0.280	5.14 (1.22)	3.24 (1.34)	**< 0.001**	3.45 (1.65)	2.94 (1.42)	0.110
Non-relative	4.22 (1.55)	4.70 (1.12)	0.473	3.57 (1.76)	3.38 (1.09)	0.780	5.66 (1.02)	5.24 (1.24)	0.464	4.39 (1.59)	3.26 (1.81)	0.198	4.31 (1.53)	3.28 (1.70)	0.220
Cause of death															
Trauma/accidental/non-accidental injuries	4.11 (1.13)	4.30 (1.29)	0.772	2.56 (1.21)	3.06 (1.18)	0.418	4.96 (1.65)	5.42 (0.97)	0.425	4.08 (1.39)	3.21 (1.45)	0.258	3.76 (1.88)	2.92 (1.59)	0.341
Non-communicable disease/ communicable disease	3.92 (1.45)	4.06 (1.20)	0.640	3.89 (1.65)	2.91 (1.33)	**0.005**	5.31 (1.19)	5.44 (1.00)	0.644	5.12 (1.28)	3.24 (1.40)	**< 0.001**	3.46 (1.68)	3.00 (1.47)	0.207
Unspecified/unknown	3.97 (0.904)	3.24 (1.61)	0.289	3.78 (1.71)	3.27 (2.14)	0.656	5.48 (0.981)	5.87 (0.416)	0.520	5.19 (1.19)	3.53 (2.15)	0.080	3.77 (1.58)	3.07 (0.643)	0.471
Time passed since death															
≤1 year	4.07 (0.791)	3.98 (1.51)	0.849	3.58 (1.73)	3.07 (1.49)	0.443	5.85 (0.615)	5.20 (1.16)	0.106	4.93 (1.20)	3.51 (1.66)	**0.025**	3.98 (1.75)	3.07 (1.89)	0.243
>1 year	3.91 (1.43)	4.10 (1.19)	0.501	3.78 (1.66)	2.95 (1.31)	**0.009**	5.13 (1.26)	5.51 (0.92)	0.099	5.03 (1.30)	3.18 (1.36)	**< 0.001**	3.44 (1.64)	2.98 (1.37)	0.155
Age at death															
≤ 20	3.74 (1.16)	4.38 (1.55)	0.435	3.13 (1.95)	3.33 (1.40)	0.842	5.40 (1.33)	5.87 (0.722)	0.467	4.83 (1.26)	2.65 (1.53)	**0.022**	3.07 (1.37)	3.37 (1.87)	0.757
21–40	4.55 (1.36)	4.09 (1.45)	0.571	3.80 (1.93)	3.31 (1.16)	0.579	5.54 (1.03)	5.03 (1.16)	0.398	4.80 (1.62)	3.23 (1.73)	0.120	5.00 (1.52)	3.27 (1.95)	0.099
41–60	4.86 (1.04)	3.94 (0.911)	0.056	4.03 (0.965)	2.47 (0.895)	**0.002**	4.88 (0.834)	5.31 (1.12)	0.370	5.03 (0.834)	3.41 (1.40)	**0.006**	3.67 (0.861)	3.07 (1.26)	0.260
> 60	3.68 (1.24)	4.03 (1.29)	0.252	3.86 (1.66)	3.02 (1.47)	**0.030**	5.36 (1.29)	5.52 (0.924)	0.559	5.12 (1.37)	3.28 (1.41)	**< 0.001**	3.32 (1.78)	2.87 (1.43)	0.262

Note: Two-sided *p*-values reported, Mann-Whitney U. Bold numbers are statistically significant

On the Fear of Death domain, significant differences were found between genders for the overall sample (females = 4.17 ± 1.26; males = 3.61 ± 1.21, *t (164)* = - 2.41, *p* = 0.017). In particular, UofT females scored significantly higher on this domain than males (t (83) = -2.11, *p* < = 0.037) (not in table).

In relation to death avoidance scores, females from AGU scored significantly higher than females from UofT (t (127) = 3.46, *p* < 0.001). In terms of within-group differences, AGU females scored significantly higher on this domain than AGU males (t (79) = 2.38, *p* = 0.020) (not in table). In relation to age, death avoidance scores were higher for AGU than UofT students among those in the ≥ 25 age group (t (98) = 3.07, *p* = 0.003). Death avoidance scores were higher for AGU students than for UofT students who reported losing a loved one (t (113) = 2.76, *p* = 0.007) and who reported losing a relative (t (95) = 2.88, *p* = 0.005). Regarding cause of death, AGU students scored significantly higher on this domain (t (76) = 2.87, *p* = 0.005) than UofT students when the cause of death was due to a noncommunicable or communicable disease. Finally, death avoidance was also higher when losing someone more than a year ago for AGU students (t (88) = 2.66, *p* = 0.009) and reported age at death to be between 41 and 60 years old (t (17) = 3.61, *p* = 0.002) and over 60 (t (66) = 2.22, *p* = 0.030).

Males and females from AGU compared to their counterparts from UofT scored significantly higher on approach acceptance (t (36) = 2.63, *p* = 0.013; t (127) = 7.38, *p* < 0.001, respectively). In addition, approach acceptance scores were significantly higher among both age groups (< 25 and ≥ 25) for AGU students (t (66) = 4.67, *p* < 0.001; t (97) = 6.04, *p* < 0.001, respectively). In this domain, there was a significant difference in scores related to religious observance, suggesting that participants who had answered yes in relation to the degree of observance had the highest score in approach acceptance, which decreased as the degree of religious observance decreased for each subsample (AGU: F (2,78) = 10.13, *p* < 0.001; UofT: F (2,83) = 22.45, *p* < 0.001). Among AGU and UofT students who reported a desire for religious observance, AGU students scored significantly higher on approach acceptance in both groups (t (82) = 2.00, *p* = 0.048; t (45) = 3.39, *p* = 0.001, respectively). AGU students also scored significantly higher than UofT students on this domain, among those who reported loss of a loved one (t (112) = 7.04, *p* < 0.001) or not (t (51) = 3.24, *p* = 0.002) and those who reported losing a relative (t (94) = 7.23, *p* < 0.001). Wherein the cause of death was due to noncommunicable or communicable disease, AGU students scored significantly higher on this domain than UofT students (t (76) = 6.15, *p* < 0.001). Approach acceptance scores were also significantly higher for AGU students in relation to time passed since death if reported more than a year ago (t (87) = 6.47, *p* < 0.001), and within one year (t (22) = 2.40, *p* = 0.025). Finally, on this domain, scores were significantly higher for AGU students who reported age at death to be ≤ 20 (t (10) = 2.70, *p* = 0.022), between 41 and 60 years old (t (17) = 2.89, *p* = 0.006) and over 60 (t (66) = 5.42, *p* < 0.001).

Escape acceptance scores were significantly higher in relation to the < 25 age group among AGU compared to UofT students (U = 364.0, z = -2.63, *p* = 0.009). Finally, among students who reported no religious observance, AGU students scored significantly higher than UofT students (t (33) = 3.23, *p* = 0.003) on this domain.

### Medical school training and curriculum in relation to death and grief

Most of the students from UofT (74%) indicated that they were not taught about death or grief thus far in their medical school curriculum. In comparison, 46% of students from AGU said that they were taught this during their medical school curriculum (*χ2* (1) = 6.77, *p* = 0.009) (Table 1). Of the students who reported learning about death and grief at UofT, 35% were in their third or fourth year of study, while 64% of AGU students reported that they were taught about death and grief in year 6. Most AGU students reported learning about grief during their clerkship years (*n* = 24), and 14 reported learning about grief during their pre-clerkship years. Among UofT students, 19 reported learning about how to deal with grief and death in medical school during pre-clerkship, compared to 10 during clerkship.

## Discussion

In the present study, the knowledge, and attitudes of medical school students from two culturally distinct institutions were compared to determine differences in death attitudes and CG scores due to bereavement. Over three-quarters of the study participants were females in both medical schools. This diversity has been reported globally, with more women enrolling in medical schools [35]. AGU students scored higher on CG than UofT students. The following death attitude domain scores: death avoidance, approach acceptance, and escape acceptance were likewise greater among AGU students than among UofT students. These results highlight the importance of addressing this subject in medical education and offer insightful information on the knowledge and attitudes of medical students toward loss.

As has been reported [13], most of the medical students (69%) experienced the loss of a loved one, with a slightly higher percentage among UofT students (71%) than their AGU (65%) counterparts. Of those who encountered such a loss, grandparents were the main ones whom they lost, followed by another relative. The prevalence of CG was also significantly higher among AGU students compared to UofT students warranting clinical assessment. Moreover, AGU students had higher CG scores compared to UofT students in relation to demographic characteristics, including both genders, age, higher degree of religious observance, losing a relative, death of more than one year ago, and age at death. This can be partly explained by having slightly more AGU students who experienced loss within the last year and a young deceased (≤ 20 years). Nonetheless, it is worth noting that all AGU students follow the Muslim faith, which emphasizes the importance of exercising patience in the aftermath of losing a loved one.

In the Islamic faith, death is an inescapable reality, and it is expected that adherents of the faith accept it with grace and equanimity [36–38]. In addition, it also calls on commended practices for the dead, such as praying for them, donating money in their honor, and other commendable behaviors, which keep those who are left always in the mind of those whom they are left behind [36]. Culture affects mourning and grief, and there is great variation in the expectations of the behaviors of the bereaved [39]. Islamic societies encourage the bereaved to resume their normal lives soon after the three days of mourning are over. Arab Muslim culture calls upon restraint of grief emotions, particularly among men [38]. Coping with loss occurs through the acceptance of God’s will and restraining overt grief behaviors. Furthermore, grief counseling and grief support groups that are common in Western countries almost do not exist in GCC countries.

Although there are cultural differences with respect to death and dying, it is worth noting that CG scores also differed based on the degree of religious observance, with those who were more religious having higher CG scores among AGU students. The literature shows conflicting results when examining the relationship between prolonged grief and religious belief, suggesting religiosity to be a significant predictor of prolonged grief following bereavement in some studies [40, 41] while not in others, among community samples [42, 43]. Studies also suggest that CG may be related to spirituality as a construct distinctly measured by religion [42]. Evidence also suggests that a discrepancy between the meaning of death and one’s worldview may influence an individual’s grief [42]. A study found that bereavement may lead to questions about religious beliefs if these two concepts do not align [44]. Thus, it may help those who are struggling with CG find meaning in death to help them better process bereavement.

In terms of death attitudes, in line with a study of medical interns in China, scores of escape acceptance in this study were the lowest compared to all other dimensions within each subsample for both AGU and UofT students [29]. While this may indicate that students are not viewing the notion of death as a means of alleviating their pain in their current lives, the significant difference in scores between the student sample populations suggest the need for interventions to improve the mental health status of medical students. There were significant gender differences in the domains of death avoidance and approach acceptance, such that females from AGU scored higher on death avoidance, while both males and females from AGU scored significantly higher on approach acceptance compared to UofT males and females. This is interesting because although AGU students may have a high degree of acceptance, which may be linked to cultural and religiosity differences, they are more inclined to avoid thoughts about death, particularly females. As for UofT females scoring higher than their male counterparts on fear of death, the diversity of the UofT students’ cultural backgrounds makes it difficult to explain.

The act of avoiding or evading the topic of death may be a defensive or coping mechanism that helps to relegate the concept of mortality to an individual’s subconscious mind; however, research suggests that females may be more prone to acknowledging these thoughts than men [28].

The approach acceptance attitude implies belief in a happy afterlife [33]. It has been well documented that belief in an afterlife is related to religious beliefs and practices. Research suggests that individuals with strong religious commitments were both more likely to believe in an afterlife and to show less fear of death [45, 46]. Our findings align with such studies that found that religious belief was linked to higher scores in accepting death [29]. In this study, although there were significant differences in relation to approach acceptance scores among the student samples, strong or moderate religious observance was associated with higher scores on this domain for both samples. This finding may also be because medical students may have a greater level of impartial acknowledgment of mortality and demonstrate a lower than usual level of fear toward it [29].

Female students scored significantly higher on the dear of death domain than males. In a study conducted in China [29], exploring the correlation between gender and attitude toward mortality in a group of Chinese university students, their findings revealed that women exhibit a greater degree of apprehension toward death compared to men. Several factors relating to societal and cultural beliefs could account for the notion that women experience greater anxiety about death, such as societal expectations around gender roles and behaviors, as well as differences in the ways that men and women cope with stress and anxiety. For example, women may be more inclined to openly acknowledge and communicate their emotions and fears, which might create the impression that they are more fearful of death than men. In general, studies have found that death education needs are higher among female students [30]. Research suggests that female students are more receptive to learning about death than their male counterparts and display a greater inclination to perceive death as an inherent occurrence [28, 30].

Experiencing fear toward death can make it more challenging to cope with grief. A recent investigation discovered that individuals who feared death had more persistent symptoms of grief following the loss of a loved one compared to those who had come to terms with death [47]. The fear of death among healthcare practitioners may also hinder their ability to communicate effectively with patients and their families who are facing end-of-life situations. Providing education and guidance on topics related to mortality can have a positive impact on mitigating an individual’s fear of death and encouraging medical students to approach the subject in a more objective and rational manner [30]. Given the limited number of students in this study indicating that they had not learned about how to deal with death or grief during medical school, further educational interventions are needed.

Most of the students gained their knowledge in the clerkship phase. Almost three-quarters of students from UofT indicated that they were not taught about death or grief thus far in their medical school curriculum, compared to 54% of AGU students. This variation can be explained by the differences in the year of study and curricula of the two medical schools and is in line with what has been reported that medical students learn about this issue from watching physicians and during clinical encounters [3]. Overall, most of the medical school students in each group reported that their curriculum lacked the ability to address grief and how to deal with the challenges of facing death. This finding is aligned with recent research emphasizing the need for death education training among physicians [48, 49], as studies have reported a lack of education surrounding end-of-life training among medical students [50].

Future studies may want to explore the perspectives of medical students on what is most effective for them in coping with death and grief. Medical schools should consider their curricula so that students are aware of the possible experiences and emotions involved with the death of a patient, and students should be given advice on whom to turn to for support. These initiatives are also important to consider outside the lives of their medical professions, as medical students may be particularly vulnerable to the emotional trauma of facing the death of a loved one, which may be compounded by the emotional exhaustion, stress, and sociocultural pressures of the medical culture, with limited time and consideration to grieve.

The study’s findings have significant ramifications for medical training and education. Given that over one-third of the students had CG, the importance of addressing grief and bereavement in medical education and training is emphasized, as it has a significant impact on medical students’ health and wellbeing [29]. This can involve teaching students how to communicate effectively, to empathize with others, and to take care of themselves, as well as including grief and end-of-life care classes in the medical curriculum. The study also highlights the importance for medical practitioners to understand how their cultural and religious beliefs affect how they see death and bereavement.

A larger sample size, participants from various colleges and nations, and the use of mixed-methods approaches can give researchers a more complete knowledge of how medical students deal with loss and sorrow. Investigating the efficacy of programs designed to enhance medical students’ understanding of and attitudes regarding loss and grief can be helpful in enhancing medical education. To gain a more complete knowledge of medical students’ experiences, it can be helpful to examine the effects of personal loss experiences, family history of illness, and cultural and religious practices on attitudes about death and sorrow. Studies that follow subjects over time might evaluate how their attitudes regarding death and grief develop. Finally, research into how loss and grief affect medical professionals’ mental health and wellbeing can shed light on how crucial it is to help them cope with loss and grief in the healthcare setting.

### Strengths and limitations

To our knowledge, this is the first study in the Arab region comparing the grief and bereavement of medical students with those in a Western society, hence addressing a notable limitation of previous research. The study contrasted medical students from two different populations, giving important insights into disparities in knowledge and attitudes toward loss caused by culture and gender. Furthermore, the BGQ and the DAP-R, both validated questionnaires, were utilized in the study to gather information on knowledge and attitudes toward loss. The study’s findings underscore the importance of educating medical students about loss and have significant ramifications for medical education, as well as supporting those bereaved among them. The study has some limitations, as only 168 participants made up the study sample, which may restrict how broadly the results can be applied. Self-reported data, which are susceptible to social desirability bias and might not accurately represent real behavior, were used in the study. The study was carried out at two universities; therefore, it might not be representative of medical students around the world. Other variables, such as family history of illness or cultural norms that might have affected students’ attitudes regarding loss, were not considered in the study. Another limitation of this study is the potential for participation bias due to the incentive structure. Students from the UofT sample were offered a $15 gift card for completing the survey, while AGU students did not receive any incentive. This could have influenced the motivation to participate. Despite this limitation, the sample size obtained for both universities still allowed valuable insights into group differences. Future studies should aim for consistency in compensation across groups to minimize any selection bias or confounding factors related to incentives.

## Conclusions

AGU students, females, and participants who indicated a higher level of religious observance at both institutions scored higher on CG scores. Furthermore, AGU students outperformed UofT students in the categories of death avoidance, approach acceptance, and escape acceptance. These findings underline the necessity of including loss and grief in medical education so that medical specialists can provide patients and their families with the best possible end-of-life care. The study also highlights how cultural and religious beliefs influence medical students’ attitudes toward death and bereavement. Future research should examine the significance of including loss and grief in medical education and training, as well as the efficacy of initiatives designed to enhance medical students’ awareness of and attitudes toward loss and grief.

### Electronic supplementary material

Below is the link to the electronic supplementary material.

Supplementary Material 1

## Data Availability

Data and materials are available upon request from the corresponding author.

## Abbreviations

AGU: Arabian Gulf University
Analysis of Variance: ANOVA
BGQ: Brief Grief Questionnaire
CG: Complicated grief
GCC: Gulf Cooperation Council
PBL: Problem-based learning
REDCap: Research Electronic Data Capture
SD: Standard deviation
